# Dr. Thomas Lothrop’s Opinion and Dissent

**Published:** 1884-05

**Authors:** 


### Dr. Thomas Lothrop's Opinion and Dissent.

The apologetic tone of the above editorial, on the question of the publication in the Journal of the proceedings of the Erie County Medical Society, would compromise the rather positive position of the writer, in case it should go forth without explanation. How it is possible for the "two editors connected with the Niagara University" to be impartial, when their relations to the profession at large, and the legal status of the Niagara Medical College, with which they are identified, are maliciously assailed, is a problem of difficult solution. Medical men are not devoid of bias on ethical questions, or on matters of personal or professional interest. The writer has never been able to follow to its conclusions that train of reasoning which leads the mind to settled convictions on both sides of the question at issue. His colleague seems an adept at this fallacious system of logic. We doubt whether the conclusions reached are satisfactory to him, and we are confident they do not subserve the great interests of the college which honors him with a chair, at a critical moment when enemies are assailing its reputation with a view to its destruction, and at the same time impugning the motives of its founders.

Passing these personal considerations, which influence the writer in this dissent, he begs to call attention to the principle by which the Censors pretend to be actuated in their pseudo-investigation. The great question of the day in professional circles is higher medical education. The Niagara Medical College is fully committed to this principle. Its curriculum is as complete as that of Harvard or the University of Pennsylvania. It requires a preliminary examination for admission to its classes, and three full courses of lectures before admitting the student to its honors. It submits its work to an independent board of examiners, thus divorcing the teaching and graduating powers, anticipating the aim and purpose of the State Medical Faculty Bill, which the JOURNAL has warmly endorsed. The high standing and character of its faculty give assurance that this standard will be fully maintained. What principle is involved in the opposition which the Censors have raised? 1. That the responsibilities of a medical college are "of the highest order." 2. That "the restrictions are in no manner onerous or difficult of fulfillment." Supporting the Censors in these two propositions were the entire faculty of the Buffalo Medical College. How has this body met these responsibilities and observed statutory restrictions? What effort have they made to raise the standard of requirements for graduation? Opposition from this source could not have arisen from any special anxiety that the new college would do any poorer work than they have done in the education of medical men. The *casus belli* is not, then, one of principle, but lies, rather, in the ESTABLISHMENT and VERY EXISTENCE of the new college.

We congratulate, however, the medical profession in this county that two of the five members of the Board of Censors declined to sign the report, and we join in the feeling of conscious pride that there were thirty-five righteous men present at that meeting who were actuated by principle, rather than by prejudice, inspired by professional interest or personal animosity.

T. L.

NOTE.—The right of dissent to editorial opinion which each member of the staff enjoys has been greatly exceeded by Dr. Lothrop in the above. We permit its publication, however, as showing how hard it is for even good men "to refrain from words," or even to keep solemn pledges under provoking circumstances. We renew, however, our promise to our readers that the JOURNAL will continue to represent the profession. There will be no suppression of important society proceedings or other medical news for mere personal or party reasons. We do not believe that the suppression of the Erie County Society transactions (which our colleague desired) would "subserve the great interests of the college," etc., nor can we understand his timidity. We believe that the Niagara University can well afford publicity to any assault upon it.

